# Reliability analysis of the Chinese version of the Functional Assessment of Cancer Therapy – Leukemia (FACT-Leu) scale based on multivariate generalizability theory

**DOI:** 10.1186/s12955-017-0664-2

**Published:** 2017-05-04

**Authors:** Qiong Meng, Zheng Yang, Yang Wu, Yuanyuan Xiao, Xuezhong Gu, Meixia Zhang, Chonghua Wan, Xiaosong Li

**Affiliations:** 1 West China School of Public Health, Sichuan University, Chengdu, Sichuan 610041 China; 2 School of Public Health, Kunming Medical University, Kunming, 650500 Yunnan Province China; 3 School of Public Health, Guangdong Medical University, Dongguan, 523808 Guangdong Province China; 4 Department of Health Education and Basic Public Health, Kunming Health Education Institute, Kunming, 650034 Yunnan Province China; 5 Department of Hematology, The First People’s Hospital of Yunnan Province, Kunming, 650032 Yunnan Province China; 6 School of Humanities and Management, Guangdong Medical University, Dongguan, 523808 Guangdong Province China

**Keywords:** Acute leukemia, Chronic leukemia, Quality of life, Evaluation studies, Multivariate generalizability theory, Reliability

## Abstract

**Background:**

The Functional Assessment of Cancer Therapy–Leukemia (FACT-Leu) scale, a leukemia-specific instrument for determining the health-related quality of life (HRQOL) in patients with leukemia, had been developed and validated, but there have been no reports on the development of a simplified Chinese version of this scale. This is a new exploration to analyze the reliability of the HRQOL measurement using multivariate generalizability theory (MGT). This study aimed to develop a Chinese version of the FACT-Leu scale and evaluate its reliability using MGT to provide evidence to support the revision and improvement of this scale.

**Methods:**

The Chinese version of the FACT-Leu scale was developed by four steps: forward translation, backward translation, cultural adaptation and pilot-testing. The HRQOL was measured for eligible inpatients with leukemia using this scale to provide data. A single-facet multivariate Generalizability Study (G-study) design was demonstrated to estimate the variance–covariance components and then several Decision Studies (D-studies) with varying numbers of items were analyzed to obtain reliability coefficients and to understand how much the measurement reliability could be vary as the number of items in MGT changes.

**Results:**

One-hundred and one eligible inpatients diagnosed with leukemia were recruited and completed the HRQOL measurement at the time of admission to the hospital. In the G-study, the variation component of the patient-item interaction was largest while the variation component of the item was the smallest for the four of five domains, except for the leukemia-specific (LEUS) domain. In the D-study, at the level of domain, the generalizability coefficients (*G*) and the indexes of dependability (*Ф*) for four of the five domains were approximately equal to or greater than 0.80 except for the Emotional Well-being (EWB) domain (>0.70 but <0.80). For the overall scale, the composite *G* and composite *Ф* coefficients were greater than 0.90. Based on the *G* coefficient and *Ф* coefficient, two decision options for revising this scale considering the number of items were obtained: one is a 37-item version while the other is a 45-item version.

**Conclusion:**

The Chinese version of the FACT-Leu scale has good reliability as a whole based on the results of MGT and the implementation of MGT could lead to more informed decisions in complex questionnaire design and improvement.

**Electronic supplementary material:**

The online version of this article (doi:10.1186/s12955-017-0664-2) contains supplementary material, which is available to authorized users.

## Background

Leukemia, the first major cause of cancer-related deaths for children and adults under 35 years old, is an increasingly common health care problem in the world. It can be divided into two categories, i.e., acute leukemia and chronic leukemia, based on the natural course and the cellular differentiation degree. In China, it has been estimated that there were 75,300 new cases of leukemia and 53,400 deaths in 2015 [1]. The treatment can mitigate the clinical symptoms and improve the 1- and 5-year overall survival rates but also introduces toxicity that can offset the clinical benefit. For patients with cancer, the primary concerns include the disease symptoms, treatment toxicity, increased risk of second malignancy, long-term and late effects of treatment (e.g., fatigue), depressive mood and anxiety, reduced work efficiency and family dysfunction. These concerns can be reflected by a comprehensive and multidimensional index: the health-related quality of life (HRQOL). There is an increasing recognition of the need to assess the HRQOL when doctors administer certain types of clinical treatment [2]. However, the measurement of the HRQOL for patients with leukemia needs specific, reliable and effective instruments.

Consequently, several leukemia-specific instruments have been developed and used, which include the Life Ingredient Profile for hematologic malignancies [3], the Medical Research Council/EORTC Quality of Life Questionnaire-Leukaemia Module (MRC/EORTC QLQ-LEU [4]), an HRQOL scale for individuals with leukemia in long-term remission, the EORTC for Chronic Myeloid Leukaemia (EORTC QLQ-CML24), which contains an additional EORTC leukemia-specific module [5], and the Functional Assessment of Cancer Therapy-Leukemia (FACT-Leu) scale [6]. Among these instruments, the FACT-Leu scale was created by combining the Functional Assessment of Cancer Therapy-General module (FACT-G) [7] and a subscale made up of 17 leukemia-specific items. The original English version was developed by Northwestern University in the USA which has been working with us. The use of the FACT scale system has been officially sanctioned. To the best of our understanding, there have been no reports of the development of a simplified Chinese version of the FACT-Leu scale. Therefore, the major objective of this study was to develop a sound simplified Chinese version of the FACT-Leu scale.

Although the original English FACT-Leu scale has been tested and determined to be a valid, reliable, and efficient instrument for evaluating the leukemia-specific HRQOL in both acute and chronic disease [6], it is necessary to test the reliability and validity of the simplified Chinese version before its expanded use in the clinic, in consideration of the possible culture-sensitive characteristics of the HRQOL. Usually, researchers evaluate the reliability and validity of HRQOL scales using Classical Test Theory (CTT).A reliability and validity analysis of the simplified Chinese version of the FACT-Leu scale was performed using CTT. The Cronbach’s α coefficients of the overall scale were greater than 0.9, and the Cronbach’s α coefficients were greater than 0.80 at the domain levels. The intra-class correlation coefficients (ICC) of all domains and the overall scale were greater than 0.90, which indicates good to excellent reliability. The good convergent and discriminant validity and good construct validity were confirmed by a correlation analysis and factor analysis. The criterion-related validity was determined to be good when using the Quality of Life Instrument for Cancer Patients-Leukemia (QLICP-LE), a scale developed entirely by us, as a criterion; more detailed results will be reported elsewhere. CTT posits that an observed score is the combination of the true score plus error, and the reliability then is the ratio of the true score variance to the observed score variance. In CTT, the reliability is based on a single source of error, ignoring all other potential sources of error variance. When measurement error stems from multiple sources, CTT may be inadequate. Generalizability theory (GT) provides a comprehensive and unifying framework that goes beyond the CTT model of a single error term by allowing for the simultaneous analysis of main and interaction effect source of error variance [8]. The conventional reliability approaches in CTT are typically post hoc, that is, the measurement reliability is computed after the fact. GT, however, can be used proactively in planning better measurement protocols. This flexibility and forecasting capability is not generally provided by conventional reliability approaches such as CTT. GT subsumes other forms of reliability approaches (e.g., internal consistency reliability, interrater reliability, and intra-class correlation) and provides a comprehensive and unifying framework for assessing the measurement reliability, especially for complex measurement situations. This theory was pioneered in the educational field and has been used in medicine [9–21]. The application of GT includes the univariate generalizability theory (UGT) method and the multivariate generalizability theory (MGT) method. The MGT was initially proposed by Cronbach based on the multiple analysis of variance (MANOVA), and it is appropriate for multidimensional and complicated measurement situations [22]. The analysis and estimation process of MGT considers not only the variances (variance components), but also the covariance structure of domains. In MGT, the reliabilities of all domains are estimated simultaneously, rather than each domain in isolation [23]. The HRQOL assessment contains different domains, usually with a different set of items within each domain. Considering these multidimensional characteristics of HRQOL, the application of MGT becomes natural. Although some studies [24–30] used GT in assessing the reliability of HRQOL, none considered MGT. Therefore, the secondary objective of the current study was to evaluate the reliability of the Chinese version of the FACT-Leu scale by using MGT. The current study is expected to answer the following questions:

1. What is the reliability of the Chinese version of the FACT-Leu scale?
2. What impact does the test length (number of items) have on the reliability of the Chinese version of the FACT-Leu scale?
3. How should the number of items for every domain in the Chinese version of the FACT-Leu scale be changed to obtain better reliability in the future?

## Methods

### Translation of the simplified Chinese version of the FACT-Leu scale

The FACT-Leu scale was translated from the original English version into simplified Chinese. In the translation procedures four steps were carried out: forward translation, backward translation, cultural adaptation, and pilot-testing. In the first step, the original English version was independently translated into simplified Chinese by two translators (one an epidemiologist and the other an oncologist who specializes in leukemia) whose native language is Chinese but who are proficient in English. Then the two forward translated versions were compared by a research coordinator to find any differences. When the differences were identified, the research coordinator discussed them with the two translators until they all agreed on a reconciled Chinese version. In the second step, the reconciled Chinese version was translated into English by two back-translators who had never read the original English version. The two back-translators are both oncologist who are proficient in English. Then, the backward translation and the original English version were compared, and the reconciled Chinese version was further modified accordingly by the same coordinator. The process was repeated until the backward translation was identical or nearly identical to the original English version. After that, the items that were likely to lead to confusion or misunderstanding due to cultural differences were modified based on the results from an in-depth interview of oncologists and a focus group discussion. In the final step, a pilot test was carried out among 15 patients diagnosed with leukemia and who satisfied the eligibility criteria. They were asked to independently complete the scale acquired in the third step and then were interviewed using a questionnaire. Any item that was upsetting or difficult to understand should be marked and given appropriate suggestions during the interview. Based on the suggestions from the patients, necessary modifications were made and a final Chinese version for use in the formal measurement of the HRQOL was obtained.

### Structure of the Chinese versions of the FACT-Leu scale

The structure of the Chinese version of the FACT-Leu scale is the same as that of the original English version; it is composed of two subscales (the 27-item FACT-G and the 17-item leukemia-specific subscale). The FACT-G consists of four domains: the 7-item Physical Well-being (PWB) domain, the 7-item Social/Family Well-being (SWB) domain, the 6-item Emotional Well-being (EWB) domain and the 6-item Functional Well-being (FWB) domain. For ease of description, the leukemia-specific subscale is called the LEUS domain in this article. Each item of the FACT-Leu scale was rated on a five-level scoring system, namely, not at all, a little bit, somewhat, quite a bit, and very much. The positive items received scores from 0 to 4 points, while the negative items were designed with scoring in the reverse order.

### Study subjects

This study recruited inpatients diagnosed with leukemia at the First Affiliated Hospital of Kunming Medical University and the First People’s Hospital of Yunnan Province during October 2013 to March 2014. Eligible participants were aged 18 years or older, at least 2 months post diagnosis of any type or stage of leukemia, had a life expectancy of more than 3 months, and were able to read and understand the questionnaires. Exclusion criteria included a diagnosis of psychosis or dementia and illiteracy.

### Measurement process

The investigators (doctors, nurses and medical postgraduate students) obtained informed consents from the patients who agreed to participate in the study and met the inclusion criteria. The Chinese version of the FACT-Leu scale was used to measure the HRQOL of the included leukemia patients after the investigators explained the study and the scale. The HRQOL is a self-report measurement, so each patient was asked to answer the scale by himself or herself. The answers were immediately checked by the investigators to verify completeness. If missing items were found, the scale was returned to the patients immediately to fill in the missing items. The patients completed the 44-item FACT-Leu scale at the time of admission to the hospital.

### A multivariate generalizability theory approach

GT contains two stages: Generalizability Study (G-study) and Decision Study (D-study). The G-study serves as a “pilot” study that decomposes the variance and covariance components related to various error sources to help confirm the relationship between the measurement goal and measurement facets based on the data collected and using analysis of variance (ANOVA) or multiple analysis of variance (MANOVA). In the D-study, the information from the G-study is used for the planning of an “optimal” measurement protocol so that the best possible reliability can be achieved while balancing other factors.

In generalizability theory, a latent trait is defined by sets of conditions under which it may be observed; each set of conditions is called a facet. Usually, the latent trait is the object of measurement [31]. In this study, the HRQOL of patients is defined as the measurement goal and may be measured with different items and at different times; the items and times may be conceived as two different facets. Two or more facets can be combined in various ways, along with the definition of the objects of measurement (the persons), to define a set of observations, called the universe of assessable observations (UAO) for a given latent trait [31]. In a D study, a universe of generalization (UG), which contains those facets and conditions that a study was willing to generalize with a particular measurement procedure, is specified. Along with the specification of the UG, universe scores for the objects of measurement are defined. A universe score is defined as the expected value of the observed scores for a measurement goal over all conditions in the UG [31]. Usually, it is defined that the UG and the UAO have the same structure.

### Multivariate G-study design

As the first step, the G-study includes design, data collection, and estimation of the relevant variance-covariance components under the design conditions. In the current study, the measurement goal is the HRQOL of patients with leukemia, which is abbreviated as “*p*” in the design and the items facet (abbreviated as “*i*”) is the only facet of measurement because the HRQOL is a self-report measurement and there is no rater. The items facet is a random facet because a different set of items can be involved in each replication. The HRQOL data are unbalanced because there are unequal numbers of items within each domain. The domains facet (abbreviated as “*h*”) is treated as the fixed facet because every replication of the measurement of the HRQOL involved the same domains. It is defined that the facet *i* was nested within the facet *h*. In addition, facet *p* is completely crossed with facet *i* because every patient answers every item in the FACT-Leu scale. In the univariate G-study sense, this design is expressed as *p* × (*i* : *h*), where *p*, *i*, and *h* represent patients, items, and domains facets, respectively. The fixed domains facet and the random effect variance component design associated with each fixed domain level yield a multivariate G study design, *p* ^•^ × *i* ^∘^, with the number of levels for the fixed domains facet being *n*_*h*_. The solid circle, ·, indicates that the patients facet is crossed with the fixed multivariate variable (i.e., domain) whereas the empty circle, ∘, indicates that the items facet is nested within the fixed multivariate variable. In other words, there is a random effect *p* ^•^ × *i* ^∘^ design within each of the five fixed domains and any single item is only associated with a single domain [32]. The mathematical models of the *p* ^•^ × *i* ^∘^ design can be defined as

1 $$ {X}_{pi h}={\mu}_h+{\mu}_{ph}\sim +{\mu}_{i: h}\sim +{\mu}_{pi: h, e}\sim $$

or

2 $$ {X}_{pih}={\mu}_h+\left({\mu}_{ph}-{\mu}_h\right)+\left({\mu}_{i: h}-{\mu}_h\right)+\left({X}_{pih}-{\mu}_{ph}-{\mu}_{i: h}+{\mu}_h\right) $$

In Equation 1, *X*_*pih*_ is the observed score that results from a single observation in the *h* ^th^ domain and this score may be decomposed into an overall mean and several effects according to the analysis of variance model. In the right-hand side of Equation 1, *μ*_*h*_ means the overall mean in the *h* ^th^ domain; *μ*_*ph*_ ∼ (*μ*_*ph*_ ∼ = *μ*_*ph*_ − *μ*_*h*_) means the patient effect in the *h* ^th^ domain because *μ*_*ph*_ is the average score of all items for patient “*p*” in the *h* ^th^ domain; *μ*_*i* : *h*_ ∼ (*μ*_*i* : *h*_ ∼ = *μ*_*i* : *h*_ − *μ*_*h*_) means the item effect on the observed score in the *h* ^th^ domain because *μ*_*i* : *h*_ is the average score per person for item“*i*” in the *h* ^th^ domain; and *μ*_*pi* : *h*,*e*_ ~ means the residual effects in the *h* ^th^ domain, including the patient-item interaction effect and other effects. The FACT-Leu scale has five domains (*n*_*h*_ = 5), and the model equations for the different domains can be presented as follows:

3 $$ \begin{array}{c}\hfill (1){X}_{p i}=(1)\mu +(1){\mu}_p\sim +(1){\mu}_i\sim +(1){\mu}_{p i, e}\sim \hfill \\ {}\hfill (2){X}_{p i}=(2)\mu +(2){\mu}_p\sim +(2){\mu}_i\sim +(2){\mu}_{p i, e}\sim \hfill \\ {}\hfill (3){X}_{p i}=(3)\mu +(3){\mu}_p\sim +(3){\mu}_i\sim +(3){\mu}_{p i, e}\sim \hfill \\ {}\hfill (4){X}_{p i}=(4)\mu +(4){\mu}_p\sim +(4){\mu}_i\sim +(4){\mu}_{p i, e}\sim \hfill \\ {}\hfill (5){X}_{p i}=(5)\mu +(5){\mu}_p\sim +(5){\mu}_i\sim +(5){\mu}_{p i, e}\sim \hfill \end{array}\Big\} $$

It follows that the variance and covariance components for the population and the UAO can be grouped into three symmetric matrices: ∑_*p*_, ∑_*i*_ and ∑_*pi*_. Note that ∑_*p*_ is the variance–covariance component matrix among patients, ∑_*i*_ is the variance–covariance component matrix for items within a domain, and ∑_*pi*_ is the patient-item interaction within a domain. As all patients contribute data to all levels of domains facet but the items are nested in different levels of domains facet, ∑_*p*_ is a full matrix while ∑_*i*_ and ∑_*pi*_ are diagonal [32].

### Multivariate D-study designs

Once the variance and covariance matrix from the G-study results are available, they could be used in the D-study to estimate the variance components of the universe score and the variance components of the corresponding error to calculate the two reliability coefficients: the generalizability coefficients (*G*) and the index of dependability (*Ф*) for every domain and the overall scale. Several D-studies under the original measurement protocol and the new measurement protocols modified by changing the number of items were analyzed to understand how the measurement reliability could vary with a changing number of items.

#### Original measurement protocol

Following the method in a univariate *P* × *I* design (letters should be capitalized in the D-study) and the definition of the variance for the composite, we could define ∑_*P*_ = ∑_*p*_, ∑_*I*_ = ∑_*i*_/*n*_*ih*_^′^ and ∑_*PI*_ = ∑_*pi*_/∑_*nih*_ in the multivariate D-study. Note that ∑_*i*_/*n*_*ih*_^′^ means the *h* ^*th*^ diagonal element in ∑_*i*_ divided by the corresponding number of items (*n*_*ih*_^′^), and ∑_*pi*_/∑_*nih*_ means the *h* ^*th*^ diagonal element in ∑_*pi*_ divided by *n*_*ih*_^′^, because ∑_*nih*_ was designed to be a diagonal matrix containing the numbers of items within the levels of *h* (e.g., ∑_*nih*_ = *diag*(7, 7, 6, 7, 17) in this study).

In the *p* ^•^ × *i* ^∘^ design, the patient-item interaction within domain (*pi*:*h*) component alone constitutes the variation of the relative error, while the variation of the absolute error contains both the patient-item interaction within domain (*pi*:*h*) component and the item within domain (*i*:*h*) component. Therefore, the variance component of the relative error for every domain (*σ*_*δh*_^2^) and the variance component of the absolute error for every domain (*σ*_*Δh*_^2^) can be calculated using Equation 4 and Equation 5.

4 $$ {\sigma}_{\delta h}^2={\sigma}_{PI: h}^2={\sigma}_{pi: h}^2/{n}_{ih}^{\prime } $$

5 $$ {\sigma}_{\varDelta h}^2={\sigma}_{I: h}^2+{\sigma}_{PI: h}^2={\sigma}_{i: h}^2/{n}_{i h}^{\prime }+{\sigma}_{pi: h}^2/{n}_{i h}^{\prime } $$

Actually, in the *p* ^•^ × *i* ^∘^ design, *σ*_*δh*_^2^ equals to ∑_*PI*_ and *σ*_*Δh*_^2^ equals to the direct sum of diagonal matrices ∑_*PI*_ and ∑_*I*_. The *G* coefficient and the *Ф* coefficient are calculated using Equation 6 and Equation 7, respectively. In Equation 6 and 7, *σ*_*h*_^2^(*P*) is the variance component of the patients in the D-study, which is the diagonal elements in matrix ∑_*P*_(∑_*P*_ = ∑_*p*_).

6 $$ {G}_h={\sigma}_h^2(P)/\left[{\sigma}_h^2(P)+{\sigma}_{\delta h}^2\right] $$

7 $$ {\varPhi}_h={\sigma}_h^2(P)/\left[{\sigma}_h^2(P)+{\sigma}_{\varDelta h}^2\right] $$

In the MGT framework, not only these indexes for every domain of scale but also the corresponding composite values of these indexes for the overall scale obtained by defining a weight coefficient are important. The weight coefficient (*w*_*h*_) reflects the proportion of the total number of items in the measurement procedure that were associated with each *h* in the overall scale. It is commonly defined as *w*_*h*_ = *n*_*ih*_/*n*_*i* ·_, where *n*_*ih*_ is the number of items in the *h* ^*th*^ domain and *n*_*i* ·_ designates the total number of items in all domains of the overall scale, that is, *n*_*i* ·_ = *n*_*ih*(1)_ + *n*_*ih*(2)_ + *n*_*ih*(3)_ + *n*_*ih*(4)_ + *n*_*ih*(5)_. The weight coefficient is applied to the corresponding variance (*σ*_*h*_^2^) or covariance (${\sigma}_{h{ h}^{\prime }}$) to calculate the composite values of those indexes. For example, the composite universe score variance for overall scale (*σ*_*c*_^2^(*P*)) can be calculated by Equation 8, while the composite variance of the relative error (*σ*_*δc*_^2^) and the absolute error (*σ*_*Δc*_^2^) for the overall scale can be calculated by Equations 9 and 10, respectively.

8 $$ {\sigma}_c^2(P)={\displaystyle \sum_{h=1}^{n_h}{w}_h^2{\sigma}_h^2(P)}+{\displaystyle \sum_h^{n_h}{\displaystyle \sum_{\ne {h}^{\prime}}^{n_h}{w}_h{w}_{h^{\prime }}{\sigma}_{h{ h}^{\prime }}(P)}{w}_h^2} $$

9 $$ {\sigma}_{\delta c}^2={\displaystyle \sum_{h=1}^{n_h}{w}_h^2{\sigma}_{PI: h}^2} $$

10 $$ {\sigma}_{\varDelta c}^2={\displaystyle \sum_{h=1}^{n_h}{w}_h^2{\sigma}_{I: h}^2}+{\displaystyle \sum_{h=1}^{n_h}{w}_h^2{\sigma}_{PI: h}^2} $$

After *σ*_*c*_^2^(*P*), *σ*_*δc*_^2^ and *σ*_*Δc*_^2^ are obtained by Equations 8 ~ 10, the composite *G* coefficient and the composite *Ф* coefficient for the overall scale can be calculated by making use of the same principles as used in Equation 6 and 7. The estimated variance-covariance component matrices and all indexes used to reflect the measurement error and reliability in MGT can be calculated using a specialized software, mGENOVA. The software and the manual for mGENOVA [33, 34] can be downloaded from the website http://www.uiowa.edu/~itp or http://www.uiowa.edu/~casma. mGENOVA can process an almost unlimited number of observations very rapidly.

In this research, the execution process of mGENOVA included three steps. Step1: A database was established and saved or transformed into a text file in the same folder as the mGENOVA application on the basis of its data structure (see Additional file 1: Data Structure and a Screenshot). Step2: A code file that contains a set of control cards and those necessary parameters (such as the filename of the database, the filename of the analysis results, the name of each domain, the number of domains, the number of subjects, and the number of items in every domain) was created and saved as a text file in the same folder as the mGENOVA application. In this code file, all parameters were separated from each other by any number of spaces and the order in which the parameters were provided was fixed; they must occur in column 10 or beyond. An example of a code is shown in Additional file 2: Example Code of mGENOVA. Step3: mGENOVA.exe was double-clicked and then mGENOVA prompted us for the filename of the code file. After the filename of the code file (for example, FACTLEU.txt) and a return were typed, mGENOVA completed its execution.

#### New measurement protocols

To understand how the numbers of items would affect the measurement reliability of the Chinese version of the FACT-Leu scale and provide some suggestion for the modification of the scale, several multivariate D studies were conducted using a three-step process.

In the first step, the test length (the number of items) was varied proportionally to three test lengths: the original test length and half and double test lengths. For example, the “half” test length had a total of 24 items, where *n*_*ih*(*PWB*)_^′^ = *n*_*ih*(*SWB*)_^′^ = *n*_*ih*(*FWB*)_^′^ = 4, *n*_*ih*(*EWB*)_^′^ = 3 and *n*_*ih*(*LEUS*)_^′^ = 9 because the number of items was rounded off. It was assumed that there were 4 random parallel items each of the PWB domain, SWB domain, and FWB domain, 3 items in the EWB domain and 9 items in the LEUS domain. Based on the results of previous D studies and the criteria that an optimal reliability coefficient is usually defined as greater than 0.80, we could determine which domain should be increased and which domain should be decreased based on the number of items. Then, the range in which the number of items of every domain should be changed in the next D-study analysis was defined.

In the second step, five scenarios were designed. In each scenario, only the number of items in one domain was changed, while the other four domains kept their original test lengths. In the first scenario, scenario A, the number of items in the PWB domain was changed in the range defined in the first step, while the other four domains kept their original test lengths; the same steps were followed for scenario B, C, D and E by changing the number of items in domains SWB, EWB, FWB and LEUS, respectively. Several multivariate D-studies were conducted for each scenario to find the appropriate number of items for each domain, deemed to be the number that made either the *G* coefficient or *Ф* coefficient just above 0.8 with simultaneously observed large composite *G* and composite *Ф* coefficients (>0.90). To describe the results of every scenario more concisely, the “appropriate number of items” that made the *G* coefficient just larger than 0.8 was defined as *n*_*i*_^*G*^, and the “appropriate number of items” that made the *Ф* coefficient just larger than 0.8 was defined as *n*_*i*_^*ϕ*^.

In the third step, the numbers of items for the five domains were reallocated based on the *n*_*i*_^*G*^ for each domain and the *n*_*i*_^*ϕ*^ for each domain found in the second step to provide two decision options for the further modification of the scale, and two multivariate D-studies were then conducted for the two decision options.

## Results

### General characteristics of the patients

After excluding the patients who were not willing or unable to participate, 101 eligible inpatients diagnosed with leukemia were included. The patients were aged 18 ~ 80 years old (the mean age was 40.46 ± 15.12 years old); 55.4% were males and most (80.2%) were of the Han ethnicity. For their education level, 60.4% completed high school. 76.2% were married and most (92.1%) had public insurance. Only 10.9% thought their economic status was well-off. 69 patients (68.3%) were diagnosed with acute leukemia, and 32 (31.7%) were diagnosed with chronic leukemia. The characteristics of the patients are provided in Additional file 3: Table S1.

### Reliability based on MGT

#### G study results

As discussed previously, the MGT applications include the G-study and D-study. Table 1 presents the G-study results for all variance components (the diagonal elements) and covariance components (covariation among the domains) of the five domains of the Chinese version of the FACT-Leu scale based on the current design (7 items in PWB domain, 7 items in SWB domain, 6 items in EWB domain, 7 items in FWB domain, and 17 items in LEUS domain; 44 items in total).

**Table 1:** Estimated variance and covariance components for *p* ^•^ × *i* ^∘^design in G-study for the five domains of FACT-Leu (*n* = 101)

	PWB	SWB	EWB	FWB	LEUS
Patient(*p*)	**0.642**	0.230	0.912	0.536	0.949
	0.108	**0.343**	0.378	0.414	0.333
	0.512	0.155	**0.491**	0.534	0.976
	0.354	0.200	0.308	**0.679**	0.667
	0.434	0.112	0.391	0.314	**0.327**
Item(i)	**0.131**				
		**0.057**			
			**0.230**		
				**0.150**	
					**0.471**
Patient × Item(*pi*)	**0.668**				
		**0.542**			
			**0.949**		
				**0.874**	
					**0.949**

Diagonal elements are estimated variance components and are presented in bold type

Lower diagonal elements are covariances

Upper diagonal elements are correlations

Each of the variance components of the patients (*σ*_*p*_^2^) represented the estimated “true score” variance across the patients on the specific domain of the scale. Based on the results, this variance component was the largest (0.679) for the FWB domain, followed by the PWB domain (0.642) and the EWB domain (0.491). The variance component of the LEUS domain was the lowest (0.327). Such information suggested that, relatively speaking, the HRQOL of the patients with leukemia differed most on the FWB domain and least on the LEUS domain. The correlation coefficients between the five domains were relatively large, those between the PWB domain and EWB domain, PWB domain and LEUS domain, and EWB domain and LEUS domain, with values of 0.912, 0.949 and 0.976, respectively. These results further corroborated that it was very suitable to use MGT methods to evaluate the reliability of the FACT-Leu scale.

The sources of variation for every domain were grouped into three parts: from patient, from item and from patient-item interaction. For the PWB domain, among the three sources, the variation component of the patient-item interaction (*σ*_*pi*_^2^) was the largest, the variation component of the patient was in the second place and only a small amount of variation was due to the item. Similar results were observed for three of the other four domains (SWB, EWB, and FWB), except for the LEUS domain, in which the variation component of the item ranked second. Given that the largest source of variation in a domain score was from the person-item interaction, we can assume that different subjects might react to the same item in different ways, despite having the same total score on the scale.

#### D study results

##### (a)D-study results under the original measurement protocol

The D-study results under the original measurement protocol are presented in Table 2. It shows that the *G* and *Ф* coefficients for four of the five domains were approximately equal to or greater than 0.80, except for the EWB domain based on original test length. These two reliability coefficients for the EWB domain were greater than 0.70 but smaller than 0.80. The variance components of error when estimating the universe score by using the sample mean for all domains were smaller than 0.05. Additionally, it is clear that the *G* coefficient was larger than *Ф* for every domain.

**Table 2:** D-study results for the five domains and the overall scale for *P* ^•^ × *I* ^∘^ design based on original test length

Index	PWB (n_i_^′^ = 7)	SWB (n_i_^′^ = 7)	EWB (n_i_^′^ = 6)	FWB (n_i_^′^ = 7)	LEUS (n_i_^′^ = 17)	Overall scale^a^ (n_i_^′^ = 44)
*σ* ^2^_*P*_	0.642	0.343	0.491	0.679	0.327	0.323
*σ* ^2^_*δ*_	0.095	0.077	0.158	0.125	0.056	0.019
*σ* ^2^_*Δ*_	0.114	0.086	0.197	0.146	0.084	0.025
${\sigma^2}_{X_{PI}}$	0.026	0.012	0.045	0.029	0.032	0.009
G	0.871	0.816	0.756	0.845	0.854	0.945
*Ф*	0.849	0.800	0.714	0.823	0.796	0.928

*σ* ^2^_*P*_Universe score variance, *σ* ^2^_*δ*_ Relative error variance, *σ* ^2^_*Δ*_ Absolute error variance, ${\sigma^2}_{X_{PI}}$ Error variance for mean, *G* generalizability coefficient, *Ф* index of dependability

^a^ For the overall scale, all indexes are the corresponding composite values

Based on the weight coefficients (listed in Table 3), the variance components of the universe score and of the corresponding errors for the five domains were integrated, and then the composite *G* and the composite *Ф* were computed for the overall scale, with the results presented in Table 2. The last column in Table 2 shows that the composite *G* and composite *Ф* coefficients were both greater than 0.90 and the composite variance component of error when estimating the universe score using the sample mean was 0.009 based on the original test length.

**Table 3:** Comparison between the CRCUS and the PDS for every domain

Index	PWB	SWB	EWB	FWB	LEUS
Number of items	7	7	6	7	17
PDS/Weight Coefficient (%)	15.91	15.91	13.64	15.91	38.63
CRCUS (%)	20.37	8.27	15.76	17.72	37.88
Absolute difference between PDS and CRCUS (%)	4.46	−7.64	2.12	1.81	−0.75
Relative difference between PDS and CRCUS (%)	28.03	−48.02	15.54	11.38	−1.94

*PDS* proportion of domain score, *CRCUS* contribution rate for composite universe score, Absolute difference between PDS and CRCUS = CRCUS-PDS, Relative difference between PDS and CRCUS = (CRCUS-PDS)/PDS*100%

Further analyses (in Table 3) indicate that the contribution rate for composite universe score (CRCUS) approached the proportion of the domain score (PDS) of the original design for most domains. The greatest difference between the contributioin rate for the composite universe score and the proportion of the domain score was seen in the SWB domain (the absolute and relative differences were −7.64% and −48.02%, respectively), while the difference between these two indexes for the LEUS domain was the smallest (absolute and relative differences of 0.75% and 1.94%, respectively).

##### (b)D-study results under New measurement protocols

As mentioned in the methodology section, to provide guidance in the modification of the scale, several multivariate D-studies were conducted using a three-step process.

**Fig. 1:**
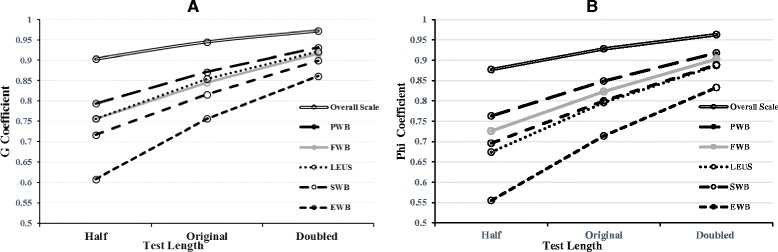
Effect on the two reliability coefficients when the test length changed. **a**: *G* coefficient **b**: *Ф* coefficient

Firstly, Fig. 1 provides a graphical representation of the effect on the two reliability coefficients (*G* and *Ф*) when the test length was changed. Figure 1 implies that a longer test length led to a larger reliability coefficient in every domain and in the overall scale. The increment speed of the two reliability coefficients from “half” to “original” was faster than that from “original” to “double”. Even though the number of items was reduced by half, the composite *G* coefficient was still greater than 0.90 and the composite *Ф* coefficient was still greater than 0.85.

As described in Table 4, among the five domains, under the original test length, both the *G* and *Ф* coefficients were the largest for the PWB domain whereas they were the smallest for the EWB domain. After the test length was reduced to a half, the *G* and *Ф* coefficients for the EWB domain were both smaller than 0.70. Given that an optimal reliability coefficient is usually defined as one that is greater than 0.80, the number of items of the EWB domain needs to be increased from the original test length, and the changing range of the number of items in the next D-study analysis was defined as from 6 to 12. In the same way, the numbers of items of the PWB domain, FWB domain, FWB domain and LEUS domain could be slightly decreased from the original test length, and the changing ranges of the numbers of items for three of the domains (PWB, SWB and FWB) were defined as from 4 to 7, while the changing ranges of the number of items for the LEUS domain was defined as from 9 to 17. Information on the reallocation of the numbers of items in the five scenarios is provided in Additional file 3: Table S2.

**Table 4:** Comparison of the two reliability coefficients for every domain and the overall scale for various test lengths

Domain	Number of items			*G* coefficient			*Ф* coefficient		
	half^b^	original^b^	double^b^	half	original	double	half	original	double
PWB	4	7	14	0.794	0.871	0.931	0.763	0.849	0.918
SWB	4	7	14	0.717	0.816	0.899	0.696	0.800	0.889
EWB	3	6	12	0.608	0.756	0.861	0.555	0.714	0.833
FWB	4	7	14	0.756	0.845	0.916	0.726	0.823	0.903
LEUS	9	17	34	0.756	0.854	0.921	0.674	0.796	0.887
Overall scale^a^	24	44	88	0.903	0.945	0.972	0.877	0.928	0.963

^a^For the overall scale, the *G* coefficient is the composite *G* coefficient and the *Ф* coefficient is the composite *Ф* coefficient

^b^half means the half test length, original means the original test length, and double means the double test length

**Fig. 2:**
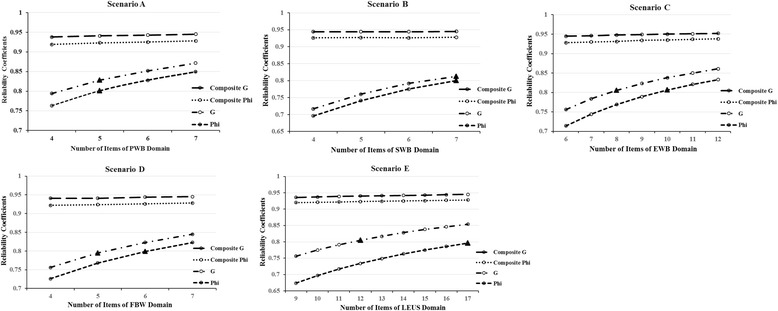
Effects on the reliability coefficients when varying the numbers of items under five scenarios. Note: The corresponding coefficients of *n*_*i*_^*G*^ and *n*_*i*_^*ϕ*^ for every domain are indicated by solid triangles

Several multivariate D-studies were conducted for each scenario designed in the second step, and the results are presented in Fig. 2. It is clear from the five panels of Fig. 2 that both the *G* and *Ф* coefficients increased with the number of items. As shown in the first panel (Scenario A) of Fig. 2, *n*_*i*_^*G*^ and *n*_*i*_^*ϕ*^ for the PWB domain were both 5, and the composite *G* and *Ф* coefficients were both greater than 0.90. Similar results were presented in the second panel (Scenario B) except that *n*_*i*_^*G*^ and *n*_*i*_^*ϕ*^ for the SWB domain were both 7. In turn, it could be seen that *n*_*i*_^*G*^ for the EWB domain was 8 and *n*_*i*_^*ϕ*^ was 10 in Scenario C of Fig. 2, *n*_*i*_^*G*^ for the FWB domain was 5 and *n*_*i*_^*ϕ*^ was 6 in Scenario D, and *n*_*i*_^*G*^ for LEUS domain was 12 and *n*_*i*_^*ϕ*^ was 17 in Scenario E.

The results of the two multivariate D-studies for the two decision options designed based on these above-mentioned *n*_*i*_^*G*^ and *n*_*i*_^*ϕ*^ for each domain are shown in Table 5. The total number of items for the overall scale in decision option A, where the numbers of items for the five domains were reallocated based on *n*_*i*_^*G*^, could be decreased from 44 to 37, while the total number of items for the overall scale in decision option B, where the numbers of items for five domains were reallocated based on *n*_*i*_^*ϕ*^, would be slightly increased from 44 to 45. It is clear that the composite *G* and *Ф* coefficients were both greater than 0.90 for both decision option A and decision option B. In decision option A,the *G* coefficients for all domains were greater than or approximately equal to 0.80, but only *Ф* coefficients for the PWB and SWB domains were greater than or equal to 0.80. In decision option B, the *G* and *Ф* coefficients for all domains were greater than or approximately equal to 0.80.

**Table 5:** Allocation of the numbers of items and the *G* and *Ф* coefficients for every domain and the overall scale for the two decision options

Decision option^b^	Index	PWB	SWB	EWB	FWB	LEUS	Overall scale^a^
Decision option A	Number of items	5	7	8	5	12	37
	*G* coefficient	0.828	0.812	0.805	0.795	0.805	0.935
	*Ф* coefficient	0.801	0.800	0.769	0.768	0.734	0.916
Decision option B	Number of items	5	7	10	6	17	45
	*G* coefficient	0.828	0.812	0.838	0.823	0.854	0.945
	*Ф* coefficient	0.801	0.800	0.806	0.799	0.796	0.928

^a^For the overall scale, *G* coefficient is the composite *G* coefficient and *Ф* coefficient is the composite *Ф* coefficient

^b^Decision option A: the best allocation of items for the overall scale based on *n*_*i*_^*G*^, Decision option B: The best allocation of items for the overall scale based on *n*_*i*_^*ϕ*^

## Discussion

### Reliability of the Chinese version of the FACT-Leu

The reliability is the extent of variation reflected by the measured results that is the result of accidental errors in the system. In other words, the reliability refers to the dependability, reproducibility, stability, and consistency of the measurement. In GT, the *G* and *Ф* coefficients are two important reliability coefficients that are used to depict the reliability for the “relative decision” and “absolute decision”, respectively. Within the GT framework, the “relative decision” depends on the norm-referenced score interpretation, which considers the consistency of the relative standings of the individuals rather than the consistency of the actual scores, while the “absolute decision” depends on criterion-referenced score interpretation which considers both the consistency of the relative standings of the individuals and the consistency of the actual scores [16]. The coefficients are selected depending on the researchers’ interests. In HRQOL research, if one’s interest lies in conducting a norm-referenced measurement to compare the HRQOL of different patients (relative decision), the *G* coefficient should be selected to specifically determine the HRQOL and quantify the dependability of the score. If one’s goal is to perform a criterion-referenced test to investigate the HRQOL of patients (absolute decision), the *Ф* coefficient should be used to inform about how dependable a score is. It is clear that the *Ф* coefficient is typically lower than the *G* coefficient for every domain because the variance component of item within domain (*i:h*) is factored into the absolute error variance and *Ф* coefficient but not the relative error variance or *G* coefficient.

Some researchers [31, 35, 36] suggested that the reliability of an instrument is generally good when the reliability coefficients (*G* coefficient or *Ф* coefficient) are greater 0.8 in GT. The composite *G* and *Ф* coefficients were greater than 0.90 for the Chinese version of the FACT-Leu scale with the two indexes for four of the five domains all approximately equal to or greater than 0.80 except for the EWB domain (greater than 0.70 but smaller than 0.80) based on the original test length. Even though the number of items was reduced to a half, the composite *G* coefficient was still greater than 0.90 and the composite *Ф* coefficient was still greater than 0.85. These results indicated a very high level of measurement reliability for the Chinese version of the FACT-Leu scale as a whole, and the measurement reliability was also good at the domain levels. Additionally, the results that the contributioin rate for the composite universe score approached the proportion of the domain score in the original design for most domains (except for the SWB domain) exemplified that the allocation of the scale items was reasonable in general. However, it is worth paying attention to the quality of the items in the SWB domain in the future because the relative difference between these two indexes in the SWB domain was the greatest and the difference value was negative.

### Influence of the number of items and suggestions for revision

The G and *Ф* coefficients increased with the number of items and were both greater than 0.8 in the double test length design. However, doubling the test length might not be realistic in practice, because it is possible that the reliability would conversely decrease with the inclusion of too many items due to an excessive consumption of time. An interesting finding in Fig. 1 was that the increment speed of the two reliability coefficients from “original” to “double” gradually slowed down. It was expected that the reliability can be increased without increasing testing time by reallocating the items to different domains. Therefore, we provided two decision options in which the best allocation of the number of items for the overall scale could be designed. Similar to the discussion above, the two decision options for further modifying the Chinese version of the FACT-Leu would be selected from depending on the researcher’s interest. If one’s interest lies in ranking (relative decision), a good consideration would be decision option A, in which the number of items in the EWB domain would increase from 6 to 8, the number of items of the SWB domain would remain the same, the numbers of items of the other three domains would be slightly reduced, and the total number of items for the scale would be 37. If one’s interest lies in the absolute standings (absolute decision), a better selection would be decision option B, in which the number of items of the EWB domain would increase from 6 to 10, the numbers of items of the SWB domain and LEUS domain would remain unchanged, the numbers of items of the other two domains would be slightly reduced, and the total number of items for the scale would be 45.

### Limitations

It is worth noting that the sample size of the study is not very large, which may affect the findings to some extent. In addition, the study subjects were from the inpatient population at hospitals, which may affect the generalizability of the scale. Therefore, larger and additional studies in which the study subjects are expanded to other populations such as outpatients are needed to validate the scale. Using MGT analysis, we put forward the suggestions about varying the number of items for every domain from the macroscopic point of view. However, we cannot answer which items would be removed by using MGT analysis. In the follow-up study, we should screen and select the items through the item response theory (IRT) methods.

## Conclusion

To sum up, the Chinese version of the FACT-Leu scale has good reliability as a whole based on the results of MGT, and the implementation of MGT could lead to more informed decisions in complex questionnaire design and improvement. If the Chinese version of the FACT-Leu scale will be modified in the future, there will be two available choices depending on the decision needed (relative decision or absolute decision) based on our analytical results: a 37-item version and a 45-item version.

## Additional files

Additional file 1: Data Structure and a Screenshot. (DOC 365 kb)

Additional file 2: Example Code of mGENOVA. (DOC 23 kb)

Additional file 3: Supplementary Results. **Table S1**. General characteristics of the patients included. **Table S2.** Allocation of the numbers of items per domain for the five scenarios. (DOC 53 kb)

## Abbreviations

ALL: Acute lymphoblastic leukemia
AML: Acute myeloid leukemia
ANOVA: Analysis of variance
CLL: Chronic lymphocytic leukemia
CML: Chronic myelogenous leukemia
CRCUS: Contribution rate for composite Universe score
CTT: Classical test theory
D-study: Decision study
EORTC QLQ-CML24: EORTC for Chronic Myeloid Leukaemia
EWB: Emotional Well-being (FACT-Leu)
FACT-G: Functional Assessment of Cancer Therapy-General module
FACT-Leu: Functional Assessment of Cancer Therapy–Leukemia
FWB: Functional Well-being (FACT-Leu)
G: Generalizability coefficients
G-study: Generalizability study
GT: Generalizability theory
HRQOL: Health-related quality of life
ICC: Intra-class correlation coefficients
IRT: Item response theory
LEUS: Leukemia-specific subscale (FACT-Leu)
MANOVA: Multiple analysis of variance
MGT: Multivariate generalizability theory
MRC/EORTC QLQ-LEU: Medical Research Council/EORTC Quality of Life Questionnaire-Leukaemia Module
PDS: Proportion of domain score
PWB: Physical Well-being (FACT-Leu)
QLICP-LE: Quality of Life Instrument for Cancer Patients-Leukemia
SWB: Social/Family Well-being (FACT-Leu)
UAO: Universe of assessable observations
UG: Universe of generalization
UGT: Univariate generalizability theory
*Ф*: Indexes of dependability
