# Supplementary material for: Reliability analysis of the Chinese version of the Functional Assessment of Cancer Therapy – Leukemia (FACT-Leu) scale based on multivariate generalizability theory
Source: Health Qual Life Outcomes. 2017 May 4;15:93. doi: 10.1186/s12955-017-0664-2 (PMC5418704; doi:10.1186/s12955-017-0664-2)
Supplement: Supplementary file 1 — Data Structure and a Screenshot. (DOC 365 kb) [file 12955_2017_664_MOESM1_ESM.doc]

**The Data Structure of the $p^{\bullet}\times i^{\circ}$ Design and a Screenshot of the Database**

The data structure of the $p^{\bullet}\times i^{\circ}$ design is showed in the following table blow. However, the data need to be inputted continuously (see a screenshot of the database in Fig. S1).

The data structure of the $p^{\bullet}\times i^{\circ}$ design

|  | *h1* | | | |  | *h2* | | | |  | *h3* | | | |  | *h4* | | | |  | *h4* | | | |
| --- | --- | --- | --- | --- | --- | --- | --- | --- | --- | --- | --- | --- | --- | --- | --- | --- | --- | --- | --- | --- | --- | --- | --- | --- |
| *p* | $i_{1}$ | $i_{2}$ | … | $n_{i}$ |  | $i_{1}$ | $i_{2}$ | … | $n_{i}$ |  | $i_{1}$ | $i_{2}$ | … | $n_{i}$ |  | $i_{1}$ | $i_{2}$ | … | $n_{i}$ |  | $i_{1}$ | $i_{2}$ | … | $n_{i}$ |
| 1 | $\times$ | $\times$ | $\times$ | $\times$ |  | $\times$ | $\times$ | $\times$ | $\times$ |  | $\times$ | $\times$ | $\times$ | $\times$ |  | $\times$ | $\times$ | $\times$ | $\times$ |  | $\times$ | $\times$ | $\times$ | $\times$ |
| 2 | $\times$ | $\times$ | $\times$ | $\times$ |  | $\times$ | $\times$ | $\times$ | $\times$ |  | $\times$ | $\times$ | $\times$ | $\times$ |  | $\times$ | $\times$ | $\times$ | $\times$ |  | $\times$ | $\times$ | $\times$ | $\times$ |
| 3 | $\times$ | $\times$ | $\times$ | $\times$ |  | $\times$ | $\times$ | $\times$ | $\times$ |  | $\times$ | $\times$ | $\times$ | $\times$ |  | $\times$ | $\times$ | $\times$ | $\times$ |  | $\times$ | $\times$ | $\times$ | $\times$ |
| … | $\times$ | $\times$ | $\times$ | $\times$ |  | $\times$ | $\times$ | $\times$ | $\times$ |  | $\times$ | $\times$ | $\times$ | $\times$ |  | $\times$ | $\times$ | $\times$ | $\times$ |  | $\times$ | $\times$ | $\times$ | $\times$ |
| $n_{p}$ | $\times$ | $\times$ | $\times$ | $\times$ |  | $\times$ | $\times$ | $\times$ | $\times$ |  | $\times$ | $\times$ | $\times$ | $\times$ |  | $\times$ | $\times$ | $\times$ | $\times$ |  | $\times$ | $\times$ | $\times$ | $\times$ |


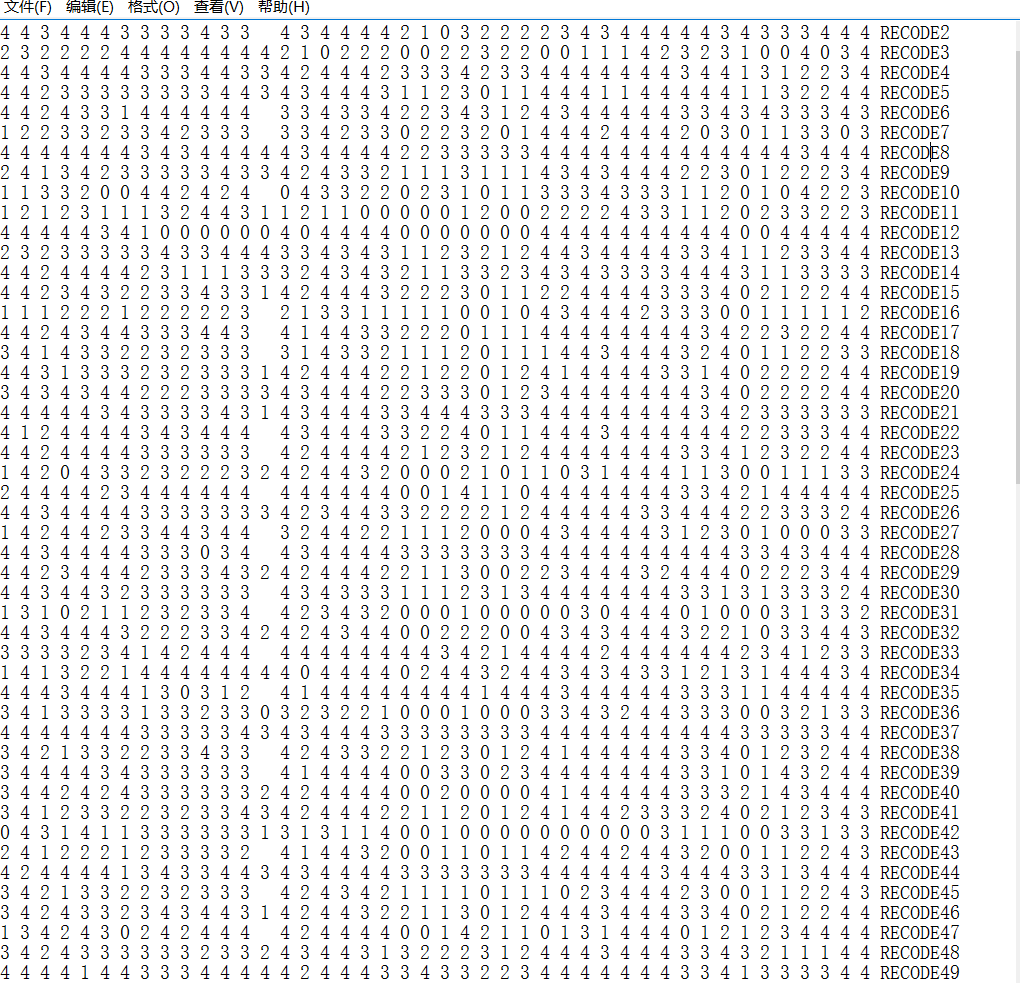


Fig. S1 A screenshot of the database:
