# Supplementary material for: Reliability analysis of the Chinese version of the Functional Assessment of Cancer Therapy – Leukemia (FACT-Leu) scale based on multivariate generalizability theory
Source: Health Qual Life Outcomes. 2017 May 4;15:93. doi: 10.1186/s12955-017-0664-2 (PMC5418704; doi:10.1186/s12955-017-0664-2)
Supplement: Supplementary file 2 — Example Code of mGENOVA. (DOC 23 kb) [file 12955_2017_664_MOESM2_ESM.doc]

**Example Code of mGENOVA various test length using** $p^{\bullet}\times i^{\circ}$**Design**

COLUMNS

123456789012345678901234567890123456789012345678901234567890123456789

GSTUDY Multivariate p3i with Covariance Components Design for FACTLEU

COMMENT FACTLEU consists of five domains each of which is rated on five dimensions

OPTIONS NRER 8"*.out" TIME DEFAULT_DSTUDY

MULT 5 PWB SWB EWB FWB LEUS

EFFECT * p 101 101 101 101 101

EFFECT i 7 7 6 7 17

FORMAT 0 3

PROCESS "FACTLEU.txt"

DSTUDY p X I Design with Covariance components Design = p*I

DEFFECT $ P 101 101 101 101 101

DEFFECT I 7 7 6 7 17

ENDDSTUDY

DSTUDY p X I Design with Covariance components Design = p

DEFFECT $ P 101 101 101 101 101

DEFFECT I 7 7 6 7 17

ENDDSTUDY

DSTUDY p X I Design with Covariance components Design = p*I

DEFFECT $ P 101 101 101 101 101

DEFFECT I 4 4 3 4 9

ENDDSTUDY

DSTUDY p X I Design with Covariance components Design = p*I

DEFFECT $ P 101 101 101 101 101

DEFFECT I 14 14 12 14 34

ENDDSTUDY

FINISH
