# Supplementary material for: Reliability analysis of the Chinese version of the Functional Assessment of Cancer Therapy – Leukemia (FACT-Leu) scale based on multivariate generalizability theory
Source: Health Qual Life Outcomes. 2017 May 4;15:93. doi: 10.1186/s12955-017-0664-2 (PMC5418704; doi:10.1186/s12955-017-0664-2)
Supplement: Supplementary file 3 — Supplementary Results. Table S1. General characteristics of the patients included. Table S2. Allocation of the numbers of items per domain for the five scenarios. (DOC 53 kb) [file 12955_2017_664_MOESM3_ESM.doc]

Table S1 General characteristics of the patients included (*n* = 101)

| Categories | Groups | *n* (%) | Categories | Groups | *n* (%) |
| --- | --- | --- | --- | --- | --- |
| Gender | Male | 56(55.4) | Insurance | Private insurance | 8(7.9) |
|  | Female | 45(44.6) |  | Public insurance | 93(92.1) |
| Ethnicity | Han ethnicity | 81(80.2) | Marital status | Married | 76(74.2) |
|  | Minority ethnicities | 20(19.8) |  | Single/Divorced | 23(23.8) |
| Education level | Primary school | 16(15.8) |  | Unknown | 2(2.0) |
|  | High school | 61(60.4) | Economic status | Poor | 43(42.6) |
|  | Junior college | 9(8.9) |  | Moderate | 47(46.5) |
|  | Above an undergraduate degree | 15(14.9) |  | Well | 11(10.9) |
| Profession | Worker | 21(20.8) | Type of leukemia | Acute | 69(68.3) |
|  | Farmer | 33(32.7) |  | ALL | 18 |
|  | Teacher | 5(5.0) |  | AML | 51 |
|  | Public servants | 2(2.0) |  | Chronic | 32(31.7) |
|  | Business owner | 7(6.9) |  | CLL | 12 |
|  | student | 6(5.9) |  | CML | 16 |
|  | Others | 27(26.7) |  | Other | 4 |

ALL, acute lymphoblastic leukemia; AML, acute myeloid leukemia;

CLL, chronic lymphocytic leukemia; CML, chronic myelogenous leukemia

Table S2 Allocation of the number of items for the five domains in the five scenarios

| Scenario | PWB | SWB | EWB | FWB | LEUS |
| --- | --- | --- | --- | --- | --- |
| Scenario A | 4~7 | 7 | 6 | 7 | 17 |
| Scenario B | 7 | 4~7 | 6 | 7 | 17 |
| Scenario C | 7 | 7 | 6~12 | 7 | 17 |
| Scenario D | 7 | 7 | 6 | 4~7 | 17 |
| Scenario E | 7 | 7 | 6 | 7 | 9~17 |
